# Role of Nanoparticle–Polymer Interactions on the Development of Double-Network Hydrogel Nanocomposites with High Mechanical Strength

**DOI:** 10.3390/polym12020470

**Published:** 2020-02-18

**Authors:** Andrew Chang, Nasim Babhadiashar, Emma Barrett-Catton, Prashanth Asuri

**Affiliations:** Department of Bioengineering, Santa Clara University, Santa Clara, CA 95053, USA; ahchang@scu.edu (A.C.); nbabhadiashar@scu.edu (N.B.); ebarrettcatton@scu.edu (E.B.-C.)

**Keywords:** hydrogel mechanical properties, nanocomposites, double-network hydrogels, polymer–nanoparticle interactions

## Abstract

Extensive experimental and theoretical research over the past several decades has pursued strategies to develop hydrogels with high mechanical strength. Our study investigated the effect of combining two approaches, addition of nanoparticles and crosslinking two different polymers (to create double-network hydrogels), on the mechanical properties of hydrogels. Our experimental analyses revealed that these orthogonal approaches may be combined to synthesize hydrogel composites with enhanced mechanical properties. However, the enhancement in double network hydrogel elastic modulus due to incorporation of nanoparticles is limited by the ability of the nanoparticles to strongly interact with the polymers in the network. Moreover, double-network hydrogel nanocomposites prepared using lower monomer concentrations showed higher enhancements in elastic moduli compared to those prepared using high monomer concentrations, thus indicating that the concentration of hydrogel monomers used for the preparation of the nanocomposites had a significant effect on the extent of nanoparticle-mediated enhancements. Collectively, these results demonstrate that the hypotheses previously developed to understand the role of nanoparticles on the mechanical properties of hydrogel nanocomposites may be extended to double-network hydrogel systems and guide the development of next-generation hydrogels with extraordinary mechanical properties through a combination of different approaches.

## 1. Introduction

Unique properties of hydrogels have enabled their use in a wide range of applications in biotechnology, bioengineering, and medicine [1,2]. Hydrogels have been used as drug delivery vehicles as they can encapsulate hydrophilic drugs and release them at a controlled rate within the body, through solute diffusion, or matrix swelling or degradation [3]. Researchers have also taken advantage of the ability of hydrogels to swell or shrink in response to external stimuli (e.g., pH, temperature) to develop biosensors for the detection of biomolecules [4,5]. Additionally, their highly porous and hydrated polymer structure mimics the extracellular cellular matrix and renders them highly suitable for in vitro cell culture, and several studies have demonstrated the successful use of hydrogels to encapsulate mammalian cells in a 3D physiological-like environment and develop in vitro models of cell proliferation, migration, and differentiation [6,7,8,9,10]. However, hydrogels have poor mechanical strength, which limits their broad applicability for tissue engineering [11]. For example, hydrogels cannot mimic stiffer tissues, which hinders their use for orthodontic or orthopedic applications [12,13]. Recent research has delved into several methods to improve mechanical properties of hydrogels. One approach that has gained significant interest over the past couple of decades is the incorporation of nanoparticles, which can enable the formation of additional crosslinks within the polymer network and contribute to enhancements in the mechanical strength of hydrogels. It has been shown that nanoparticle-mediated physical crosslinking can complement chemical crosslinking and that the combination of chemical and physical crosslinking can lead to enhancements in an elastic modulus that is greater than with either alone [14,15,16]. However, previous work suggests that there might be an upper limit to enhancements in mechanical strength achievable through the addition of nanoparticles, possibly due to the existence of a saturation point in the gains achievable through a combination of chemical and physical crosslinking [15,17,18]. Another method to improve the mechanical strength of hydrogels is to create hybrid hydrogels by combining two different polymers with contrasting properties [19]. The resulting double-network (DN) hydrogel has been shown to possess improved mechanical strength, due to the contrasting network structures and strong interpenetrating network entanglement [20,21]. Interestingly, researchers have also shown that these approaches may be combined and that the mechanical strength of DN hydrogels can be further improved with the addition of nanoparticles [22,23].

Our study sought to explore the role of polymer–nanoparticle interactions on the properties of DN hydrogel nanocomposites, and investigated if the hypotheses previously developed to understand the role of nanoparticles on the mechanical properties of (single-network) hydrogel nanocomposites may be applied to DN hydrogel systems. Specifically, rotational rheological measurements and swelling experiments were conducted to investigate if the observed saturation in nanoparticle-mediated enhancements in mechanical properties for single-network hydrogel systems also extend to double-network hydrogels. Double-network hydrogels composed of chemically crosslinked polyacrylamide (pAAm) as the primary network, and incorporating silica nanoparticles (SiNPs) were used as the model system. Previous studies have demonstrated that strong interactions between polyacrylamide chains and silica may facilitate the formation of SiNP-mediated pseudo crosslinks within the hydrogel network and mediate mechanical reinforcement of pAAm hydrogels [24,25]. Consistent with previous investigations of DN hydrogel nanocomposites, our data shows significant enhancements in the elastic modulus of DN hydrogels, as well as decreased swellability, upon the addition of nanoparticles. Our experiments also reveal that in order to observe the strongest impact of nanoparticles on the mechanical properties of DN hydrogels, both networks in the hydrogel must have strong physical adsorption to the nanoparticles. Finally, a saturation in the gains in the elastic modulus afforded by a combination of adding a second network and incorporation of nanoparticles was observed. In summary, these results indicated that nanoparticle-mediated enhancements in hydrogel mechanical properties may be a general phenomenon and similar theories may be used to describe the role of nanoparticles to improve the mechanical properties of both single- and double-network hydrogels.

## 2. Materials and Methods

### 2.1. Materials

Materials for preparation of the hydrogels, acrylamide (40% *w/v*), acrylic acid (sodium salt), n,n′-methylenebis(acrylamide) (2% *w/v*), alginic acid (sodium salt, low viscosity), ammonium persulfate (APS), n,n,n′,n′-tetramethylethylenediamine (TEMED), and calcium chloride (CaCl_2_) were purchased from Sigma Aldrich (Saint Louis, MO, USA), and agarose (low melting) was purchased from Thermo Fisher Scientific (Waltham, MA, USA), and used as received. Tris–HCl buffer (pH 7.2) was obtained from Life Technologies (Carlsbad, CA, USA) and binzil silica nanoparticle colloid solution with mean particle size of 4 nm was obtained as a gift from AkzoNobel Pulp and Performance Chemicals Inc. (Marietta, GA, USA).

### 2.2. Polymerization Reaction

All hydrogel samples were prepared using an acrylic mold (1.6 mm thick and 6.5 mm in radius) at room temperature as previously described [17,24]. The polymerization reactions were performed between parallel plates of the mold to minimize exposure to air as oxygen inhibits the free radical polymerization reaction for pAAm and poly(sodium acrylate) (pNaAc), as well as to maintain consistency in sample size across experiments. For pAAm and pNaAc samples, the monomer (AAm or NaAc) and crosslinker (Bis) stocks were diluted to their desired concentrations in pH 7.2, 250 mM Tris–HCl buffer, followed by the addition of TEMED (0.1% of the final reaction volume) and 10% w/v APS solution (1% of the final reaction volume). Agarose hydrogels were prepared by first dissolving agarose in Tris–HCl buffer at 70 °C. The agarose stock solutions were then cooled down to and maintained at 37 °C before diluting to desired concentrations and adding to the acrylic molds to form the hydrogels. Alginate gels were prepared by first dissolving alginate in Tris–HCl buffer at room temperature. The alginate stock solutions were then diluted to the desired concentrations and added to the acrylic molds, followed by the addition of 100 mM CaCl_2_ in Tris–HCl buffer to crosslink the alginate and form the hydrogels. For DN hydrogels composed of pAAm and pNaAc, NaAc monomer was added to the pAAm reaction mixture prior to the addition of APS and TEMED. Similarly, DN hydrogels composed of pAAm and alginate were prepared by adding alginate solutions to the pAAm reaction mixture prior to the addition of APS, TEMED, and CaCl_2_. For DN hydrogels composed of pAAm and agarose, the pAAm reaction mixture was first warmed to 37 °C, before the addition of agarose solutions at 37 °C and subsequent addition of APS and TEMED. For nanocomposite hydrogels, various amounts of silica nanoparticles were added to the reaction mixture prior to the addition of APS and TEMED (and CaCl_2_ for hydrogels made using alginate).

### 2.3. Rheological Measurements

Rheological measurements of neat hydrogels and hydrogel nanocomposites were carried out using the MCR302 rotational rheometer (AntorPaar, Austria), as described previously [17]. After waiting for 1 h to ensure complete gelation (gelation usually occurs within 20 min), the hydrogel discs were taken from the acrylic mold and transferred onto the lower plate of the rheometer. The samples were gently wiped with tissue paper to remove any excess water before lowering the top plate. The elastic modulus was then determined at 1 Hz and 1% strain and reported as an average of three independent measurements.

### 2.4. Measurement of the Swelling Properties

To study the swelling properties of neat hydrogels and hydrogel nanocomposites, the hydrogel disks prepared as described above were wiped with tissue paper to remove any excess water, weighed and then immersed in pH 7.2, 100 mM Tris–HCl buffer. Samples were withdrawn from the buffer at different time intervals (6, 12, and 24 h) and their weights were determined after first blotting excess buffer with tissue paper. The swelling ratios at different time intervals were calculated using the following equation:

$$Swelling\;ratio\;\left(\%\right)=\;\left[\frac{W_{t}-\;W_{0}}{W_{0}}\right]\;\times \;100\;$$

where *W_t_* is the weight of the hydrogel samples after a given time interval and *W_0_* is the initial weight (i.e., before immersing in buffer).

## 3. Results

### 3.1. Development and Characterization of the Model

**Figure 1:**
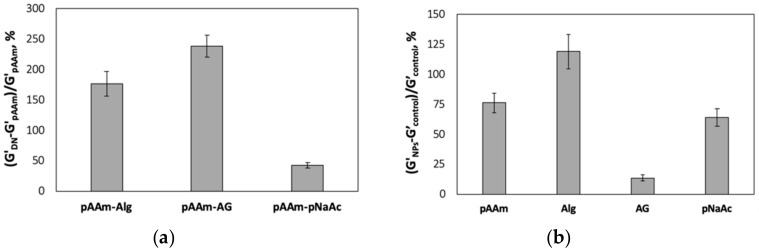
(**a**) Percent relative elastic moduli of pAAM-alginate (pAAM-Alg), pAAm-agarose (pAAM-AG), and pAAm-pNaAc DN hydrogels, prepared using 5% pAAM and 2% second polymer. Values for relative elastic modulus were calculated by normalizing the values for the DN hydrogels to pAAm hydrogels, and (**b**) percent relative elastic moduli of pAAM, alginate (Alg), agarose (AG), and pNaAc hydrogel nanocomposites prepared using 2% monomer and 2% 4 nm SiNPs. Values for relative elastic modulus were calculated by normalizing the values for the hydrogel nanocomposites to those for neat hydrogels not containing SiNPs. Data shown are the mean of triplicate measurements ± standard deviation and have been repeated at least three times with similar results.

To study the effect of SiNPs on the mechanical properties of DN hydrogels, a model system was first established using 5% *w*/*v* of pAAm as the primary network and 2% *w*/*v* of either agarose, alginate, or pNaAc as the second network. All these materials are well characterized, commercially available, and routinely used in a wide variety of industrial and scientific applications [26,27,28,29]. Moreover, they represent hydrogels that are formed through different crosslinking mechanisms: pAAm and pNaAc (free-radical copolymerization), agarose (thermosensitive sol-to-gel transition), and alginate (physical crosslinking). Characterization of the DN hydrogels using rotational rheometry revealed that the relative elastic modulus of pAAm hydrogels improved upon the addition of a second polymer network (Figure 1a). Moreover, consistent with previous investigations [20,21], DN hydrogels prepared by incorporating polymers with properties different to those of pAAm lead to better enhancements. However, it is important to note that pNaAc gels were significantly softer that alginate and agarose gels at similar concentrations (Figure S1 in Supplementary Materials), which may have an impact on the elastic modulus of DN hydrogels prepared by incorporating pNaAc as one of the network polymers.

The elastic moduli of the individual (or single-network) hydrogels (2% *w*/*v*) prepared with and without SiNPs were also measured using rotational rheology (Figure 1b). Unsurprisingly, the degree of nanoparticle-mediated enhancements was different for different hydrogels, with the highest benefit observed for alginate hydrogels. These differences in enhancements may be attributed to the differences in the extent to which SiNPs may interact with the polymer chains and contribute to the extent of crosslinking in the polymer network. For instance, while the remarkably low improvements in modulus for agarose due to the addition of SiNPs were unanticipated, this result may be explained by a no to low level of interactions between silica and agarose (to the best of our knowledge, there are no published studies that clearly demonstrate positive interactions between agarose and silica nanoparticles).

### 3.2. Influence of Chemical Crosslinking on Nanoparticle Mediated Enhancements of Hydrogel Elastic Modulus

**Figure 2:**
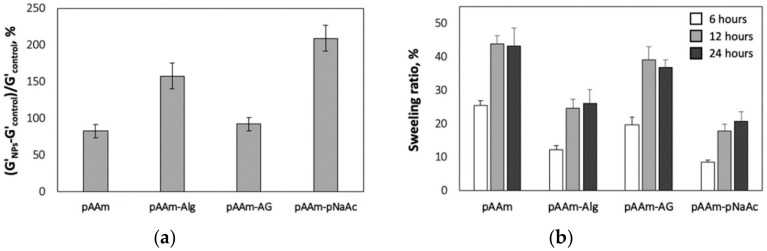
(**a**) Percent relative elastic moduli of pAAm, pAAM-alginate (pAAM-Alg), pAAm-agarose (pAAM-AG), and pAAm-pNaAc hydrogels, prepared using 5% pAAM, 2% second polymer (for DN hydrogel samples) and 2% 4 nm SiNPs. Values for relative elastic modulus were calculated by normalizing the values for the hydrogel nanocomposites to those for neat hydrogels not containing SiNPs, and (**b**) percent swelling ratios of pAAm, pAAM-alginate (pAAM-Alg), pAAm-agarose (pAAM-AG), and pAAm-pNaAc hydrogel nanocomposites, prepared using 5% pAAM, 2% second polymer (for DN hydrogel samples) and 2% 4 nm SiNPs at various time points—6 h (white bars), 12 h (light grey bars), and 24 h (dark grey bars). The values for swelling ratio were calculated by normalizing the values obtained for hydrogel samples at various time points to those obtained at time = 0 min. Data shown are the mean of triplicate measurements ± standard deviation and have been repeated at least three times with similar results.

The impact of incorporating nanoparticles on the mechanical properties of DN hydrogels was then assessed using rotational rheology, which revealed increases in elastic moduli for DN hydrogel nanocomposites relative to neat DN hydrogels not incorporating SiNPs (Figure 2a). Furthermore, it was interesting to note that the observed enhancements in elastic modulus were consistent with the observations made for single-network hydrogel nanocomposites. For instance, pAAM-agarose DN hydrogels did not significantly benefit from the addition of nanoparticles, at least not beyond the relative enhancements observed for pAAm-SiNP composites. This suggested that both hydrogels within the DN hydrogel should have strong interactions with the nanoparticles to realize the full extent of benefits afforded by the incorporation of nanoparticles.

Previous studies have indicated that nanoparticles facilitate enhancements in hydrogel elastic modulus by serving as pseudo crosslinkers and increasing the extent of crosslinking within the hydrogel network [17,25]. To check if the aforementioned differences in the enhancements in modulus for different DN hydrogel nanocomposites can be explained using a similar mechanism, swellability of DN hydrogel nanocomposites was measured. If hydrogel-nanoparticle interactions contribute to an increase in the DN hydrogel modulus through increases in the average crosslinking density, the swellability of DN hydrogel nanocomposites prepared using alginate or pNaAc should be lower than that of pAAm-agarose nanocomposites. As expected, these experiments showed decreased levels of relative swellability for pAAm-NaAc and pAAm-alginate nanocomposites compared to that for pAAm-agarose nanocomposites. Furthermore, consistent with the hypothesis and measurements of elastic modulus, the values for relative swellability of pAAm-agarose and pAAm nanocomposites were comparable. Together, these results indicated that the lack of enhancements in the elastic modulus of pAAm-agarose nanocomposites relative to pAAm nanocomposites can be explained by limited interactions between agarose and SiNPs, which dilutes the positive impact of nanoparticles on the modulus of DN hydrogels prepared using agarose.

### 3.3. Influence of Polymer–Nanoparticle Interactions on Enhancements in DN Hydrogel Modulus

**Figure 3:**
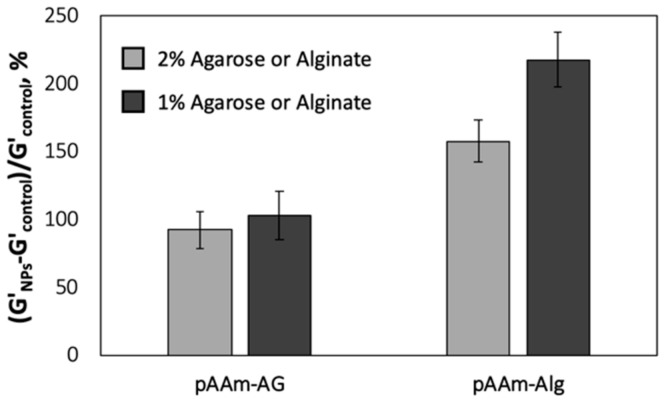
Relative elastic moduli of pAAm-agarose (pAAM-AG) and pAAM-alginate (pAAM-Alg) hydrogel nanocomposites, prepared using 5% pAAM, 2% (light grey bars) or 1% (dark grey bars) second polymer (agarose or alginate), and 2% 4 nm SiNPs. Values for relative elastic modulus were calculated by normalizing the values for the hydrogel nanocomposites to those for neat hydrogels not containing SiNPs. Data shown are the mean of triplicate measurements plus standard deviation and have been repeated at least three times with similar results.

Next, experiments were designed to further validate that the nanoparticles must exhibit strong physical interactions with both polymers in the DN hydrogel network to realize the strongest impact of nanoparticles on the DN hydrogel modulus. The enhancements afforded by the incorporation of SiNPs were compared for DN hydrogels prepared using high (2% *w*/*v*) and low (1% *w*/*v*) amounts of agarose or alginate (Figure 3). These experiments demonstrated a dependence of concentration of the second network on SiNP-mediated enhancements for DN hydrogels incorporating alginate, but not for DN hydrogels incorporating agarose. It is important to note that the elastic moduli of both pAAm-agarose and pAAM-alginate DN hydrogels increased with increasing concentration of the second polymer (Figure S2). These results offered additional evidence in support of previous observations that indicate the importance of strong polymer–nanoparticle interactions on the enhancements in DN hydrogel modulus. More importantly, diminishing impact of SiNPs on the DN hydrogel modulus at higher concentrations of alginate (Figure 3) suggested the existence of a saturation point in gains in hydrogel modulus achievable through a combination of incorporating a second polymer network and addition of nanoparticles.

**Figure 4:**
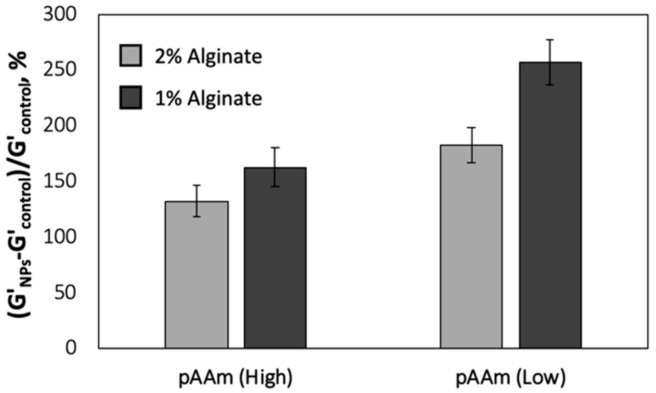
Relative elastic moduli of pAAM-alginate hydrogel nanocomposites, prepared using 5% (High) or 2.5% (Low) pAAm, 2% (light grey bars) or 1% (dark grey bars) alginate, and 2% 4 nm SiNPs. Values for relative elastic modulus were calculated by normalizing the values for the hydrogel nanocomposites to those for neat hydrogels not containing SiNPs. Data shown are the mean of triplicate measurements plus standard deviation and have been repeated at least three times with similar results.

To validate our observations that suggest a saturation point in the overall hydrogel modulus mediated by combining different polymers and incorporation of nanoparticles, the mechanical characterization studies were repeated for pAAm-alginate hydrogels and nanocomposites over a range of pAAm and alginate concentrations (Figure 4 and Figure S3). Not surprisingly, both pAAM and alginate concentrations played an important role on SiNP-mediated enhancements in the modulus of pAAm-alginate DN hydrogels. The decreasing role of nanoparticles with increasing polymer concentrations observed in this study, for pAAm-alginate DN hydrogels, was consistent with previous studies that demonstrate a decreased impact of nanoparticles on the hydrogel modulus at higher monomer concentrations [15].

## 4. Discussion

The aim of this study was to investigate the role of polymer–nanoparticle interactions on the mechanical properties of DN hydrogels using three different double-network hydrogel compositions. pAAm was used as the primary network and the second network was either alginate, agarose, or pNaAc, each one representing hydrogels with different mechanism of gelation and chemical properties. SiNPs were chosen as a model nanoparticle, given its ability to positively interact with pAAm and serve as a pseudo crosslinker to enhance the extent of crosslinking in the polymer network [25]. Results from the experimental analyses demonstrated the ability of nanoparticles to improve the mechanical properties of DN hydrogels, consistent with previous studies. However, significant enhancements in elastic modulus upon incorporation of SiNPs were only observed for pAAm-pNaAc and pAAM-alginate DN hydrogels, but not for pAAm-agarose. The rheological characterization studies were well supported by swelling studies that showed that the swellability of pAAm-pNaAc and pAAM-alginate hydrogel nanocomposites were significantly lower than that of pAAm-agarose hydrogel nanocomposites. These results suggested that the degree of improvement was not universal, and seemingly limited by the ability of nanoparticles to strongly interact with and contribute to additional crosslinking for both the polymers in the DN hydrogel network. Our experiments also revealed an inverse correlation between the polymer concentrations of DN hydrogels and the degree of nanoparticle-mediated enhancements in mechanical properties, i.e., DN hydrogels composed of higher concentrations of polymers benefited less due to the addition of nanoparticles. Further characterization of the DN hydrogel nanocomposites using morphology and microstructure analysis will be necessary to further confirm our observations, but are outside the scope of this investigation.

Overall, the results presented in this paper have two main outcomes. From an application standpoint, development of orthogonal strategies to enhance the elastic modulus is important to realize the broad applicability of hydrogels, especially in applications related to the development of stiff tissue mimics. Furthermore, the introduction of nanomaterials into the hydrogel network may allow the consideration of DN hydrogels for new applications; for example, researchers have already demonstrated the utility of DN hydrogel nanocomposites for applications in self-healing, shape memory, 3D printing, and dye removal [30,31,32]. Second, from a fundamental standpoint, this work provides a mechanistic insight into the role of nanoparticles in the mechanical properties of DN hydrogels, including elastic moduli and swellability. Our results indicated the existence of a ‘global’ saturation point for DN hydrogel nanocomposites, beyond which it becomes less plausible to enhance elastic modulus by simply increasing the concentration of the second network hydrogel or nanoparticles. A similar phenomenon previously reported for single-network hydrogels describes that gains in elastic modulus achievable through the addition of nanoparticles maybe limited by the saturations in the combined crosslinking density (i.e., the sum of chemical crosslinker- and nanoparticle-mediated crosslinking densities) [15]. Further experimental analyses may be necessary to fully elucidate the role of nanoparticles in DN hydrogels; however, our study indicates that the mechanistic understandings previously derived using single-network hydrogel systems may be applied to guide the design of DN hydrogel nanocomposites with high mechanical strengths.

## Supplementary Materials

The following are available online at https://www.mdpi.com/2073-4360/12/2/470/s1, Figure S1: Elastic modulus for neat alginate, agarose, and pNaAc hydrogels prepared using 2% monomer, Figure S2: Elastic moduli of pAAM-alginate and pAAm-agarose hydrogels, prepared using 5% pAAM and 2% or 1% alginate or agarose, Figure S3: Elastic moduli of pAAM-alginate hydrogels, prepared using 5% or 2.5% pAAm and 2% or 1% alginate.
