# Supplementary material for: Role of Nanoparticle–Polymer Interactions on the Development of Double-Network Hydrogel Nanocomposites with High Mechanical Strength
Source: Polymers (Basel). 2020 Feb 18;12(2):470. doi: 10.3390/polym12020470 (PMC7077622; doi:10.3390/polym12020470)
Supplement: Supplementary file 1 [file polymers-12-00470-s001.pdf]

polymers

Communication

Role of Nanoparticle–Polymer Interactions on the Development of Double-Network Hydrogel Nanocomposites with High Mechanical Strength

Andrew Chang, Nasim Babhadiashar, Emma Barrett-Catton and Prashanth Asuri *

Department of Bioengineering, Santa Clara University, Santa Clara, CA 95053, USA; ahchang@scu.edu (A.C.); nbabhadiashar@scu.edu (N.B.); ebarrettcatton@scu.edu (E.B.-C.)

* Correspondence: asurip@scu.edu; Tel.: +1408-551-3005

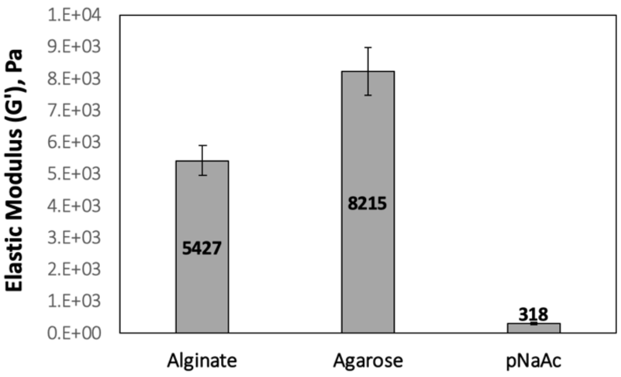

Figure S1. Elastic moduli of neat alginate, agarose, and pNaAc hydrogels prepared using 2% monomer. Data shown are the mean of triplicate measurements ± standard deviation and have been repeated at least three times with similar results.

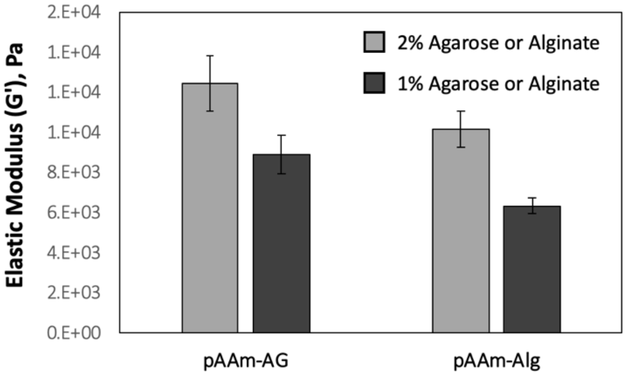

Figure S2. Elastic moduli of pAAm-agarose (pAAM-AG) and pAAM-alginate (pAAM-Alg) hydrogels, prepared using 5% pAAM and 2% (light grey bars) or 1% (dark grey bars) second polymer (agarose or alginate). Data shown are the mean of triplicate measurements plus standard deviation and have been repeated at least three times with similar results.

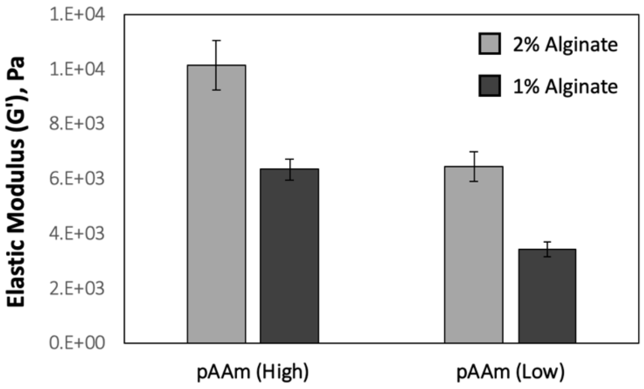

Figure S3. Elastic moduli of pAAM-alginate hydrogels, prepared using 5% (High) or 2.5% (Low) pAAm and 2% (light grey bars) or 1% (dark grey bars) alginate. Data shown are the mean of triplicate measurements plus standard deviation and have been repeated at least three times with similar results.
